# Physical-activity interventions to reduce fear of falling in frail and pre-frail older adults: a systematic review of randomized controlled trials

**DOI:** 10.1007/s41999-024-00944-9

**Published:** 2024-02-27

**Authors:** Ioannis Savvakis, Theodoula Adamakidou, Christos Kleisiaris

**Affiliations:** 1 Department of Nursing, Faculty of Health Sciences, Hellenic Mediterranean University, Heraklion, Greece; 2 Department of Nursing, Faculty of Health Sciences, University of West Attica, Athens, Greece

**Keywords:** Fear of falling, Older adults, Frailty, Physical activity

## Abstract

**Background:**

Frailty in older adults leads to progressive deterioration of their physical condition and makes them prone to develop Fear of Falling (FoF). Physical-activity interventions appear to be effective in managing the components of frailty but there is no clear evidence to determine whether physical-activity may affect FoF in frail and pre-frail older adults.

**Objective:**

Τhis systematic literature review aims to synthesize evidence on the relationship between the physical interventions to ameliorate balance, strength, and mobility and FoF reduction in frail and pre-frail older adults.

**Methods:**

Studies assessing physical-activity interventions for frail and pre-frail older adults aged 60 years and older were identified in English through searches in PubMed, ScienceDirect, and Cochrane Central Register of Controlled Trials databases till February 2023. Study quality was assessed, and a qualitative synthesis of results was performed.

**Results:**

A total of 13 studies published were included. All of them were Randomized Control Trials and the most frequent assessment tool used to assess FoF (10 of 13 studies) was the Fall Efficacy Scale-International (FES-I). Six studies were assessed as having a low risk of bias. Cumulatively, the findings of this review indicate that physical-activity interventions are effective in reducing the FoF of frail and pre-frail older adults.

**Conclusion:**

The results are encouraging and recapitulate the positive role of physical interventions in FoF reduction. However, future research would benefit from longer follow-up periods, longer intervention duration, and participation of interdisciplinary teams.

**Supplementary Information:**

The online version contains supplementary material available at 10.1007/s41999-024-00944-9.

## Key summary points

**Aim:**

The aim was to synthesize evidence on the relationship between physical-activity interventions and FοF reduction in frail and pre-frail older adults.

**Findings:**

Muscle strengthening, balance improvement, and mobility training are effective in reducing the FoF of frail and pre-frail older adults.

**Message:**

Physical-activity interventions have a positive role in FoF reduction while improving the quality of life of frail and pre-frail older adults and promoting healthy aging.

**Supplementary Information:**

The online version contains supplementary material available at 10.1007/s41999-024-00944-9.

## Introduction

Frailty is a multifactorial geriatric condition characterized by the reduction of physiological reserve such that the ability to cope with every day or acute stressors is limited. The most prevalent definition and diagnostic criteria of frailty converge on the criteria of Fried [1], who for the first time in 2001 described the phenotype of frailty. Diagnostic criteria include involuntary loss of weight and muscle mass, exhaustion, low physical activity, slowness, and reduced grip strength. The presence of three or more criteria indicates frailty while the presence of less than three pre-frailty [1]. Frailty significantly increases the risk of developing several adverse health outcomes including physical limitation (disability), hospitalization, loss of Activities of Daily Living (ADL), risk of falls, fractures, and mortality [2].

A fall is defined as ‘‘an unexpected event in which the participants come to rest on the ground, floor, or lower level” [3]. Fear of falling (FoF) is defined as ‘‘an emotional response to a real or imagined threat to balance” [4]. Fear itself constitutes a state of emotion that signifies the recognition of being in danger. The commonly used term 'fear of falling' actually signifies general concerns about the possibility of experiencing a fall [4]. It seems that these concerns develop in response to an individual becoming aware of the possibility of injury following a potential fall or from the experience of the fall itself [5]. However, most concerns are not related to the pain of an injury, but how an injury will affect their ability to continue living a fulfilling life and how it would affect the lives of those they would have to care for if they were to be hurt [5].

FoF is the term used in much of the available literature. Recently the World Falls Guidelines Concern about Falls and Falling Working Group recommended using the term ‘concern about falling’ instead of ‘fear of falling’ [6]. The complexity of terms used in the literature to describe fall-related impacts arises from their subjective conceptual meaning. These terms may include concern about falling, fear of falling, anxiety, balance confidence, and self-efficacy [7, 8]. In this review, the term fear of falling (FoF) serves as an umbrella for all subjective concepts.

FoF affects older adults’ daily routine and physical activities and may lead to restrictions on their physical capabilities, decreasing their quality of life [9–11]. About 30% of the elderly fall at least once a year and 20% of them need medical and hospital care [12]. Pre-frail and frail older adults are at an even significantly higher risk of experiencing falls and developing FoF [13] and frail older adults are at 1.8 times higher risk of falls compared to their robust counterparts [14]. Frailty in older adults leads to concern about falling and, as a result, limitation of their daily activities [15]. FoF seems to be prevalent among community-dwelling frail older adults, with a significant negative impact on their physical function, quality of life, and social interaction [16]. Frail older adults are weaker, slower, and less vigorous than their counterparts and may suffer from sarcopenia and exhaustion, resulting in avoidance of daily activities, leading to progressive deterioration of their physical condition and making them more prone to recurrent falls [13], developing FoF in a percentage ranging from 48.1 to 50.7% [17]. The more developed the FoF, the higher the level of frailty in community-dwelling older adults [18]. This knowledge of the association between FoF and frailty is important as health professionals can proactively help older people not to develop FoF by increasing their physical activity and self-confidence in a way that this vicious cycle can be stopped [18].

Serious injuries sustained by falls have a negative impact on quality of life and are associated with disability and mortality [19]. They have a profound negative impact on older adults’ morbidity, and despite the efficient surgical methods, assistance with mobility and ADL is required for long-term care [20]. FoF in older adults with hip fractures affects their rehabilitation process due to reduced consistency to physical therapy and is related to loss of confidence, consequent fear of re-falling, and depression and may lead to self-limited levels of activity, reduced physical function, and social interaction [20–22]*.*

Α recent review reported that occupational therapy interventions improve functionality and reduce FoF in community-dwelling frail older adults [23]. Thus, it is essential, that older adults especially frail and pre-frail older adults, be motivated by health professionals in active aging to prevent FoF and future falls and to remain independent with activities of daily living. Older adults must keep their physical activity level (strength, balance, mobility) in good condition to prevent loss of muscle mass and bone quality, to reduce the risk of disabilities [24], and also to reduce FoF decreasing the risk of falls [25].

However, to the best of our knowledge, no review studies have been conducted so far to determine whether physical-activity interventions reduce FoF in frail and pre-frail older adults. Therefore, this systematic review aimed to present an overview of the physical-activity interventions that effectively reduced the FoF in frail and pre-frail older adults. More specifically this systematic review synthesizes evidence on the relationship between FoF-related interventions ameliorating balance, strength, and mobility and the reduction of FoF in frail and pre-frail older adults.

## Methods

This review followed the updated guidelines of PRISMA 2020 for reporting systematic reviews [26] and synthesis without meta-analysis [27].

### Eligibility criteria

Studies were eligible for inclusion if they were published in English and involved frail and pre-frail older adults aged 60 years and older, without mental health issues, having no diagnosis of cognitive impairment (e.g., dementia), and taking no psychoactive medication. In addition, to meet the inclusion criteria, articles had to have at least one physical-activity intervention to improve strength, balance, and/or mobility and an assessment tool for the above-mentioned interventions and FoF. Articles that were commentaries, protocols or pilot studies, and feasibility studies, were excluded. Likewise, articles that were using only cognitive and social intervention were excluded. Articles that referred to chronic conditions that rendered participants incapable of independent mobility or caused severe impairment of their neuromuscular functionality were also excluded from this review.

### Search strategy

The search followed terms such as: ‘fear of falling’, ‘older adults’, ‘frail’, ‘pre-frail’, and ‘frailty’, and the terms ‘strength’, ‘balance’, and ‘mobility’ that referred to aspects of physical activity. The aforementioned terms were used in the advanced search method along with the application of filters (AND, OR, NOT). More specifically, the following pattern was applied: *(‘physical activity’ [Title/Abstract] OR ‘strength’ [Title/Abstract] OR ‘balance’ [Title/Abstract] OR ‘mobility’ [Title/Abstract]) AND (‘falls’ [Title/Abstract] OR ‘fear of falling’ [Title/Abstract]) AND (‘older adults’ [Title/Abstract] OR ‘elderly’ [Title/Abstract] OR ‘aged’ [Title/Abstract]) AND (‘frail’ [Title/Abstract] OR ‘pre-frail’ [Title/Abstract] OR ‘frailty’ [Title/Abstract]).* A filter of Randomized Control Trials was applied. An extensive electronic search was conducted in three electronic databases (PubMed, ScienceDirect, and Cochrane Central Register of Controlled Trials) till February 2023 for studies meeting the eligibility criteria using the same search strategy.

### Data collection process

All articles that were retrieved, were meticulously selected, and stored in the Mendeley software, and duplicates were excluded. The screening was made by two independent researchers. For the final inclusion, an assessment was conducted by two researchers working independently, who read the full text of the identified studies, to meet the eligibility criteria. In case of discrepancies, a third researcher took part to reach a consensus.

### Data items

Extracted data included the title, the year of publication, the study design, the characteristics of participants and their health condition, the validity and reliability of the design tools, the assessment of FoF, and the physical activity of the participants.

### Study risk of bias assessment

Two researchers independently assessed the quality of the included studies to identify any potential risk of bias. Risk of bias was evaluated using the revised Cochrane Risk-of-Bias tool (RoB 2) [28] in randomized trials. This tool is based on five domains: (1) randomization process; (2) deviation from the intended intervention; (3) missing outcome data; (4) measurement of the outcome; (5) selection of the reported results. Each domain comprises relevant questions and a judgment for each respective domain (“low risk of bias,” “some concerns,” or “high risk of bias”). The judgments within each domain lead to an overall risk-of-bias final conclusion [28].

## Results

### Study selection

**Fig. 1:**
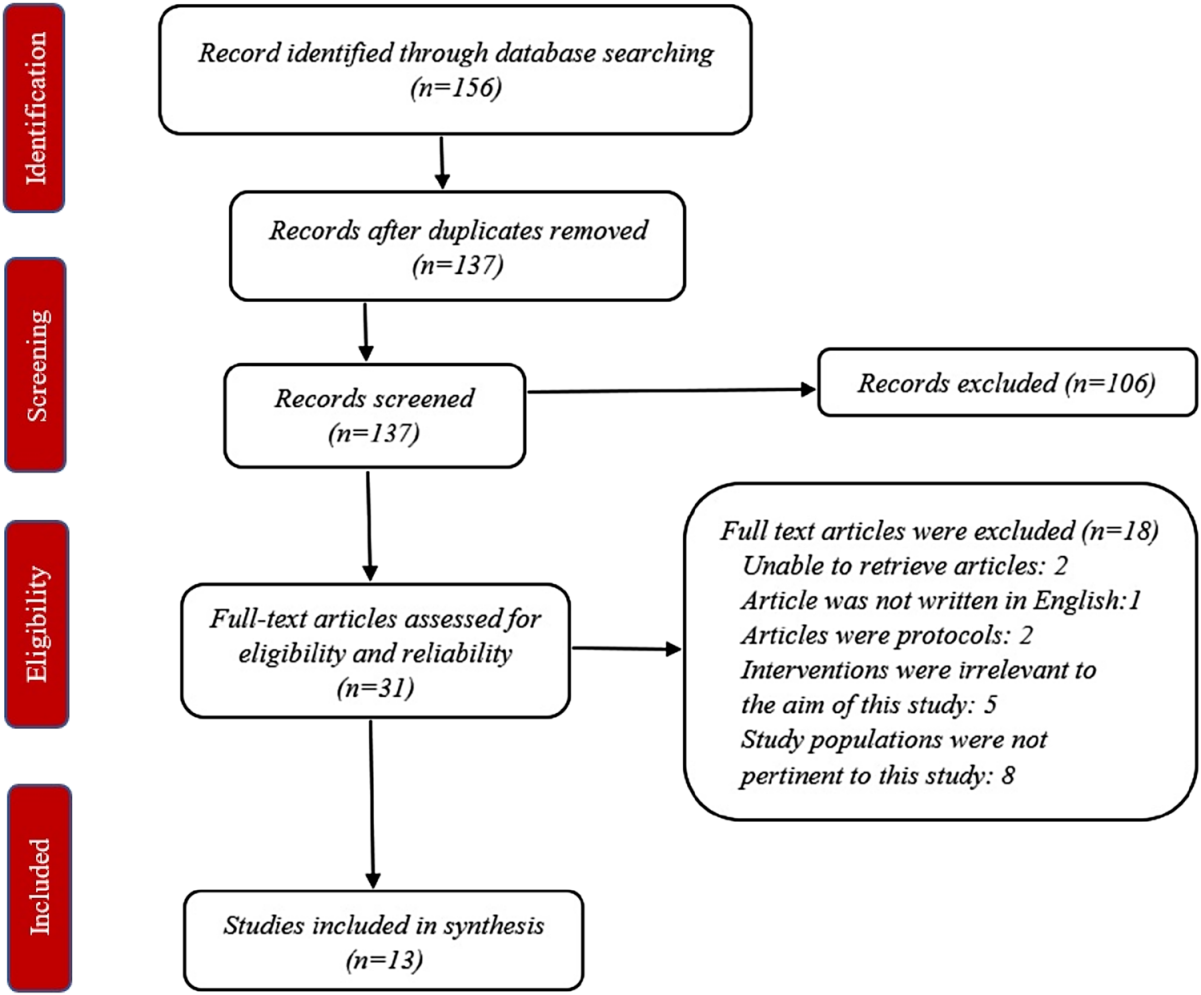
Study flow diagram (PRISMA)

The search produced 156 items that were inputted into the Mendeley software. After removing duplicate context, 137 articles were initially selected for this systematic review. 106 articles were excluded through title and abstract screening. The remaining 31 full-text articles were assessed for eligibility of which 18 were further excluded due to the following reasons: two articles were unable to retrieve, one article was not written in English, two articles were protocols, five articles had interventions that were irrelevant to the objective of this review and eight articles had study populations that were not pertinent to this review (four had study populations aged ≤ 60, three with cognitive impairments, and one did not include older adults with frailty). A total of 13 articles were included for qualitative analysis in this systematic review (Fig. 1).

### Study characteristics

Table 1 summarizes the characteristics of the included studies. Five studies were conducted in Europe, four in Asia, two in South America, and two in the USA. The vast majority of studies (*n* = 9) were Randomized Control Trials (RCTs), two were Cluster RCTs, another one was Single-blind RCT and one was a three-arm RCT.

**Table 1:** Characteristics of included studies

Studies	Study design	Sample characteristics	Frailty status assessment	Outcome assessment	Interventions
Chittrakul et al. [29], 2020 (Thailand)	RCT	*n* = 72 Aged ≥ 65 IG: *n* = 36 Pre-Frail CG: *n* = 36 Pre-Frail	Fried’s Frailty Phenotype	FES-I^a^	Multi-system Physical Exercise (MPE) program
Gomes et al. [30], 2018 (Brazil)	RCT	*n* = 30 Aged ≥ 71 IG: *n* = 15 Frail & Pre-Frail CG: *n* = 15 Frail & Pre-Frail	Fried’s Frailty Phenotype	FES-I^a^	Interactive video gaming
Pollock et al. [31], 2012 (UK)	Single-blind RCT	*n *= 77 Aged ≥ 70 IG: *n* = 38 Frail CG: *n* = 39 Frail	Not mentioned	FES-I^a^	Whole-body vibration therapy
Sattin et al. [32], 2005 (USA)	Cluster RCT	*n* = 311 Aged ≥ 70 IG: *n* = 158 Pre-Frail CG: *n* = 153 Pre-Frail	Speechley and Tinetti’s classification	ABC Scale^b^ FES-I^a^	Tai Chi exercises
Ge et al. [33], 2021 (China)	Cluster RCT	*n* = 65 Aged ≥ 60 IG: *n* = 32 Pre-Frail CG: *n* = 33 Pre-Frail	Fried’s Frailty Phenotype	3-point Likert-scale	Tai Chi exercises
Kapan et al. [34], 2017 (Austria)	RCT	*n* = 80 Aged ≥ 65 IG: *n* = 39 Frail CG: *n* = 41 Frail	Share – Frailty instrument	FES-I^a^	Strength training
Jeon et al. [35], 2014 (South Korea)	RCT	*n* = 62 Aged ≥ 65 IG: *n* = 31 Frail CG: *n* = 31 Frail	Not mentioned	FES-I^a^ 4-point Likert-scale	Education strength and balance exercises
Furtado et al. [36], 2020 (Portugal)	Three arm RCT	*n* = 60 Aged ≥ 70 CME: *n* = 21 Frail CSE: *n* = 20 Frail CG: *n *= 19 Frail	Fried’s Frailty Phenotype	FES-I^a^	Chair-multimodal and muscle-strength exercises
Moreira et al.[37], 2021 (Brazil)	RCT	*n* = 66 Aged ≥ 60 IG: *n* = 32 Pre-Frail CG: *n* = 34 Pre-Frail	Fried’s Frailty Phenotype	FES-I^a^	Interactive video gaming
Giné-Garriga et al.[38], 2013 (Spain)	RCT	*n* = 51 Aged ≥ 80 IG: *n* = 26 Frail CG: *n* = 25 Frail	Fried’s Frailty Phenotype	ABC Scale^b^	Functional circuit training (FCT) program
Yamada et al. [39], 2011 (Japan)	RCT	*n* = 307 Aged ≥ 65 Frail group: *n* = 159 Robust group: *n* = 148	Time up and Go test (TUG)	FES-I^a^	Resistance muscle strength training
Hagedorn et al. [40], 2010 (USA)	RCT	*n* = 27 Aged ≥ 70 IG: *n* = 15 Frail CG: *n* = 12 Frail	Not mentioned	FES-I^a^	Computer feedback training system and strength exercises
Sihvonen et al. [41], 2004 (Finland)	RCT	*n* = 27 Aged ≥ 70 IG: *n* = 20 Frail CG: *n* = 7 Frail	Not mentioned	Single-item question method	Individualized visual feedback-based balance training

*IG* Intervention group, *CG* Control group, *CSE* Chair Muscle Strength Exercise group, *CME* Chair-Multimodal Exercise Group

^a^FES-I assesses the level of concern about falling

^b^ABC scale assesses balance confidence

Interventions aiming at ameliorating FoF of the participants ranged between 1 month and 1 year in duration and were performed by physiotherapists [29–31], Tai Chi Chuan instructors [32, 33], trained volunteers [34], principal and assistant researcher [35], and exercise specialists [36]. The remaining five studies did not clarify who implemented the interventions.

Six studies used Fried’s Frailty Phenotype [29, 30, 33, 36–38] to assess the frailty status of older adults. One study used Speechley and Tinetti’s classification [32], another one used the Share-Frailty instrument [34] and one used the Time up and Go test (TUG) [39]. The remaining four studies did not clarify what kind of assessment tools had been used to clarify the frailty status of the participants.

The most frequent assessment tool used to assess FoF (10 of 13 studies) was the Falls Efficacy Scale—International (FES-I) [29–32, 34–37, 39, 40]. The level of concern about falling was also measured with the Activities-specific Balance Confidence (ABC) Scale in two studies, one as a main assessment tool [38] and one as a supplementary [32]. In addition, two studies used simple questions about the concern and FoF on a three- or four-point Likert scale (1 = not at all concerned to 4 = very concerned) one as a main [33] and the other as a supplementary assessment tool [35]. The remaining study [41] used the Single-item question method, which asked the subjects whether they were worried about falling. The score ranged from 0 to 3 points. The higher the score, the more afraid they were of falling.

The main FoF-related interventions included in the studies were Tai Chi Exercises [32, 33], functional training in a dynamic and static position [29, 36, 38] or on a Whole-body vibration platform [31], functional tasks using only body weight [29, 36, 38], balance exercises on a force platform [41], interactive video [30, 37] and computer feedback training [40], resistance strength exercises [35, 39, 40] and exercises with resistance elastic bands [34, 36].

### Risk of bias in studies

**Fig. 2:**
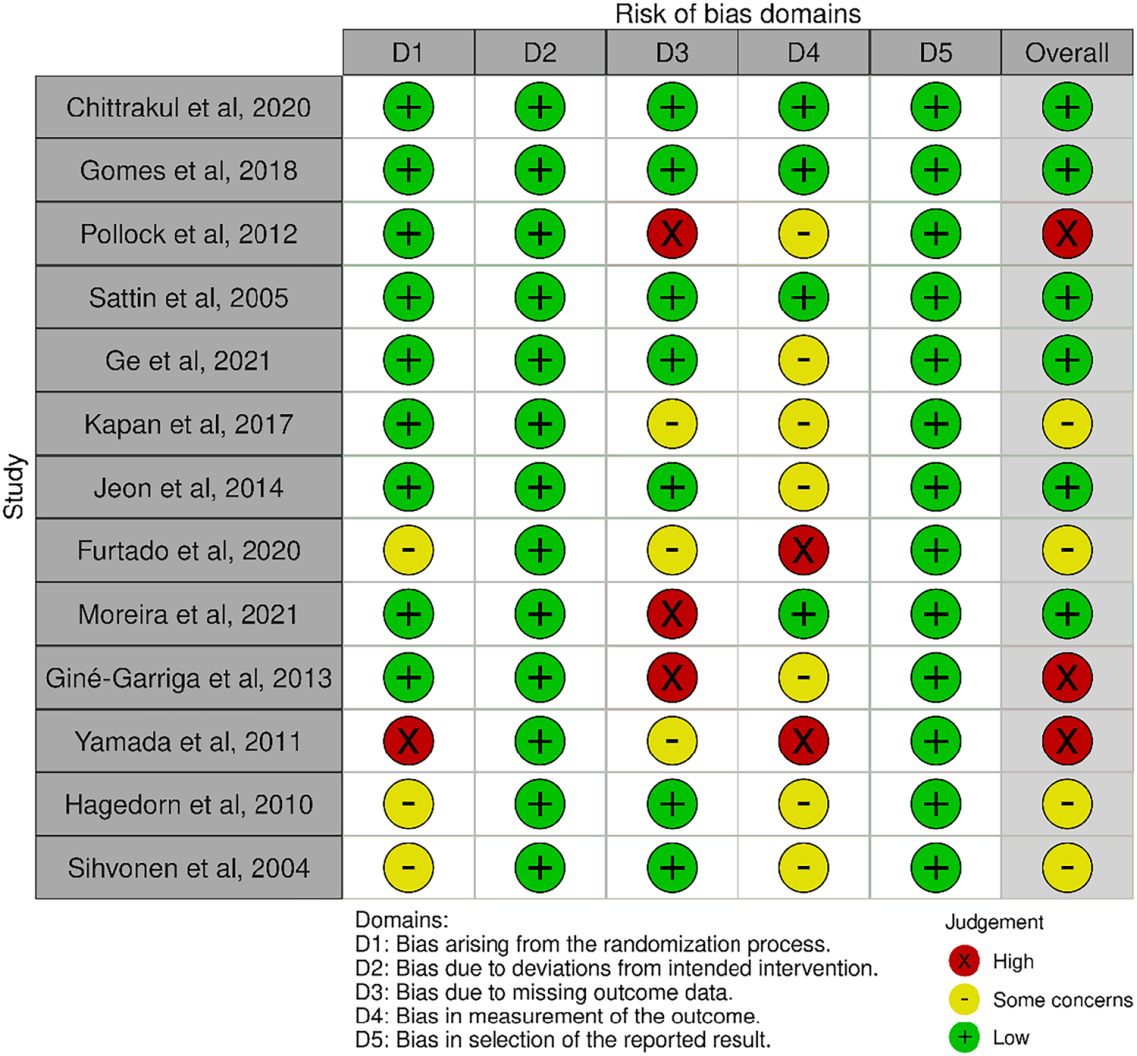
Risk of bias for each study. Cochrane risk of bias tool for randomized trials RoB 2

Six of the studies showed a low risk of bias overall [29, 30, 32, 33, 35, 37]. The randomization details were not clarified in four studies [36, 39–41]. Six studies on the third domain—missing outcome data—indicated a higher risk of bias than the others [31, 34, 36–39]. This is related to participant withdrawal during interventions. In three studies the dropout rate was over 20% [31, 37, 38]. Seven studies on the fourth domain—concerning measurement outcome—indicated some concern [31, 33–35, 38, 40, 41] and two studies a high risk of bias [36, 39]. This was mainly related to the non-reporting or the validity of the assessment tools, the non-intervention in control groups, and the lack of blinding of the assessors or the participants. In the second and fifth domains, all studies showed a low risk of bias (Fig. 2).

### Results of synthesis

The type of physical-activity interventions and their respective effects on the FoF are shown in Table 2. The available data on the impact of strength, balance, and mobility training on the FoF are analyzed below.

**Table 2:** Results of synthesis

Studies	Length of study	Interventions group			Control group (intervention)	FoF outcomes	
		Strength	Balance	Mobility		Results	Conclusion
Chittrakul et al. [29], 2020 (Thailand)	Twelve weeks	Sit-to-stand, knee raise, squats, step back lunges	Heel & toe walking, steps in multiple directions & surfaces	–	Flexibility exercises	[Fes-I (IG:18.05 vs CG:25.69, *p* < 0.001] [Fes-I (IG:24.27 vs CG:38.52, *p* < 0.001]	Significant FoF reduction after 12 and 24 weeks of follow-up
Gomes et al. [30], 2018 (Brazil)	Seven weeks	–	Interactive video gaming (to improve postural control)	Interactive video gaming (to improve gait)	Information outlining the benefits of physical activity	[Fes-I (IG:40.3 vs CG:34.7, *p* > 0.05] [Fes-I (IG:38.6 vs CG:34.5, *p* > 0.05]	No significant differences after 7 weeks and 1 month of follow-up
Pollock et al. [31], 2012 (UK)	8 weeks	Progressive strength training plus Whole-body vibration platform balance, and mobility exercises	Balance exercises plus Whole-body vibration platform	Functional mobility training plus Whole-body vibration platform	Strength, balance, and mobility exercises only	[Fes-I (IG:32.4 vs CG:30.9, *p* > 0.05] [Fes-I (IG:35.9 vs CG:37.7, *p* > 0.05]	No significant differences after 8 and 6 months of follow-up
Sattin et al. [32], 2005 (USA)	48 weeks	–	Tai Chi exercises. Coordination, narrowing of lower extremity stance	Tai Chi exercises Trunk rotation, weight shifting,	Wellness education relevant to health	[ABC IG: 59.2 vs CG: 47.9, *p* < 0.001] [Fes-I (IG:17.6 vs CG:21.2, *p* < 0.001]	Significant FoF reduction after 48 weeks of follow-up
Ge et al. [33], 2021 (China)	8 weeks	Tai Chi exercises (lower limb strength)	–	Tai Chi exercises (gait function)	Usual care	[Likert-scale (IG:0.16 vs CG:1.00, *p* < 0.001]	Significant FoF reduction after 8 weeks of follow-up
Kapan et al. [34], 2017 (Austria)	12 weeks	Mini squat, beetles, hip extensions, strength exercises with elastic band	–	–	Social support	[Fes-I (IG:39.9 vs CG:41.5, *p* = 0.016]	No significant differences after 12 weeks of follow up
Jeon et al. [35], 2014 (South Korea)	12 weeks	Muscle strengthening exercise – ankle (dorsiflexion) and lower extremity (hip, knee extension, and flexion)	Balance exercise – static (standing on one leg) and dynamic (weight shifting, walking in multiple directions)	–	No intervention	[Fes-I (IG: 7.48 vs CG: 0.49, *p* < 0.001] [Likert-scale (IG:0.23 vs CG:0.13, *p* < 0.001]	Significant FoF reduction after 12 weeks of follow-up
Furtado et al. [36], 2020 (Portugal)	28 weeks	CSE Group: elastic-band resistance exercises, squat, chest & shoulder press, hip flexion, Chair spine twist, biceps flexion & triceps extension	–	CME Group: Agility integrated exercise, Chair-based sit and reach, leg extension, skipping, walking	No intervention	[Fes-I (CSE:18.11 vs CME:32.08 CG:39.67, *p* < 0.001]	Significant FoF reduction after 28 weeks of follow-up
Moreira et al. [37], 2021 (Brazil)	12 weeks	Xbox 360 Console “Your Shape™: Fitness Evolved” game (squats and lunges)	Xbox 360 Console “Your Shape™: Fitness Evolved” game (Boxing, lateral & anteroposterior displacements)	–	Strength and balance exercises	[Fes-I (IG:23.22 vs CG:25.62, *p* = 0.37]	No significant differences after 12 weeks of follow-up
Giné-Garriga et al. [38], 2013 (Spain)	12 weeks	Functional circuit training program. Rising from a chair, stair climbing, knee bends, leg squat, leg extension & flexion, calf raise, floor transfer & lunges	Functional balance exercise—static (standing on one leg, dual tasks, tandem standing with eyes open/closed using different surfaces) and dynamic (walking on multiple surfaces & directions)	–	No intervention	[ABC IG: 64.69 vs CG: 47.63, *p* < 0.001] [ABC IG: 55.79 vs CG: 49.15, *p* < 0.001]	Significant FoF reduction after 12 and 36 weeks of follow-up
Yamada et al. [39], 2011 (Japan)	12 months	Frail group: Resistance exercise training (leg press, leg curl & leg extension)	–	–	Robust group: Resistance exercise training (leg press, leg curl & leg extension)	[Fes-I (FG:35.9 vs RG:37.1, *p* < 0.001]	Significant FoF reduction after 12 months of follow-up
Hagedorn et al. [40], 2010 (USA)	12 weeks	Progressive resistance muscle strength (leg press, pulley station, ball games)	Visual computer feedback system (weight shifts, one leg & toe standing, double tasks in different directions and surfaces)	–	Traditional balance and strength training	Fes-I (IG:24.8 vs CG:27.7, *p* > 0.05]	No significant differences after 12 weeks of follow-up
Sihvonen et al. [41], 2004 (Finland)	4 weeks	–	Individualized dynamic balance exercises on a force platform (dynamic weight shifting, leaning, stepping tasks on different surfaces, verbal tasks)	–	No intervention	[Single-item question method (60% (*n* = 12) of the IG had no FoF vs 14% (*n* = 1) of the CG, *p* = 0.02] [Single-item question method (37% (*n* = 7) of the IG had no FoF vs 14% (*n* = 1) of the CG, *p* > 0.02]	Significant FoF reduction after 4 weeks of follow-up No between-group differences were found after 12 months of follow-up

*IG* Intervention group, *CG* Control group, *FG* Frail group, *RG* Robust group, *CSE* Chair Muscle Strength Exercise group, *CME* Chair-Multimodal Exercise Group

### Strength

Ten studies included strength interventions aiming to ameliorate FoF of frail and pre-frail older adults such as resistance muscle strengthening exercises (leg press, leg curl, leg extension & flexion, hip extensions, chest & shoulder press, trunk extension, biceps flexion & triceps extension), strength exercises with an elastic band, proprioceptive and functional exercises (rising from a chair, stair climbing, knee bends, pulley station, ball games, chair spine twist), strength exercises using body weight (squat, beetles, floor transfer & lunges sit-to-stand, step back lunges), Tai Chi exercises, Whole-body vibration platform, and interactive video gaming.

The majority of studies strongly supported that different strength training means have a positive influence on improving the FoF in frail and pre-frail older adults [29, 33, 35, 36, 38, 39]. Three studies indicate a significant FoF reduction within groups but no significant differences between groups were found [31, 34, 37]. One study investigated progressive resistance strength training in improving FoF of frail older adults, but no statistically significant differences after the intervention compared to baseline were found [40].

### Balance

Nine studies included balance interventions aiming to ameliorate FoF of frail and pre-frail older adults. The procedures followed to improve balance included static and dynamic balance exercises. More specifically, standing on one leg, tandem standing with eyes open/closed using different surfaces, and standing on the bosu ball were used to improve static balance. Furthermore, to improve dynamic balance weight shifting, leaning, heel & toe walking, stepping up and down, walking in multiple directions and double tasks were used. Interactive video gaming, Visual computer feedback system, a Whole-body vibration platform, and a force platform were also used.

Four of the nine studies supported that different balance interventions have a positive impact on FoF in frail and pre-frail older adults [29, 32, 35, 38]. Two studies, though, indicate a significant difference in FoF immediately after the intervention and the follow-up within groups, but no difference in comparison to the two [31, 37]. Contrariwise, a randomized controlled trial investigated the effect of balance training in FoF with a visual computer feedback system and the authors found no effect after the intervention [40]. One study that included interactive video gaming as an intervention to improve postural control, was not able to reduce the FoF of frail and pre-frail older adults immediately after the intervention and 1 month of follow-up [30]. Another study indicates that despite the beneficial effects of a 1-month individualized dynamic balance training in FoF, follow-up measurements at 1-year post-intervention, show that training protective effects against FoF maintain decline [41].

### Mobility

Five studies included mobility interventions aiming to ameliorate FoF of frail and pre-frail older adults such as Tai Chi exercises (trunk rotation, weight shifting), functional mobility training plus Whole-body vibration platform, interactive video gaming, agility integrated exercise, chair-based sit and reach, leg extension, skipping, and walking.

Three studies supported that different mobility interventions can improve FoF in frail and pre-frail older adults [32, 33, 36]. One study indicates that despite the significant difference in FoF within groups immediately after the intervention, pointed out no significant differences between groups [31]. One study that included interactive video gaming as an intervention to improve gait, indicates no significant reduction of FoF immediately after the intervention and 1 month of follow-up [30].

## Discussion

This systematic review presents a synthesis of the evidence concerning the effectiveness of physical-activity interventions to reduce FoF in frail and pre-frail older adults. The evidence from the published articles included in this review focuses on the effects of balance, strength, and mobility improvement on FoF reduction and self-esteem build-up in both frail and pre-frail older adults. In most studies [29, 32, 33, 35, 36, 38, 39, 41], it is reported that physical-activity interventions focusing on muscle strengthening, balance improvement, and mobility training can significantly contribute to fall reduction and minimize associated fear in frail and pre-frail older adults.

The results indicate that muscle invigoration may have a positive impact on physiological mechanisms that enhance motor function in consistent with previous studies [42, 43]. It is supported that different training means and combinations with balance and/or mobility exercises, can reduce FoF [29, 33, 35, 36, 38]. It seems that frail and pre-frail elderly can benefit from a timely intervention and are still capable of carrying out intense exercises to invigorate their neuromuscular system as previous studies have shown [44, 45].

Seven studies evaluated physical activity and it appeared to increase after the exercise intervention [29, 34, 36–39, 41]. In five of these studies, FoF was significantly reduced [29, 36, 38, 39, 41]. This may indicate that interventions that helped to improve physical activity led to greater reductions in FoF. The above result is consistent with previous studies showing that exercise interventions have a positive effect on improving physical activity and reducing the fall risk in community-dwelling older people [46, 47]. It also appears that the possibility of a reduction in FoF is four times higher among those who increased their physical activity, compared to those whose physical activity did not change or decrease [34].

In some of the included studies, no differences were detected between the groups. A potential reason for this includes the limited number of participants, which might have reduced statistical power to identify significant differences [30, 31, 37, 40]. In addition, some of these studies exhibited high dropout rates which could have introduced bias and influenced the overall results, especially if those who dropped out had systematic differences from those who completed the study [31, 37]. Furthermore, there is a concern about selection bias in one study, where recruitment occurred solely through social media [34]. This in turn might have led to the participation of highly motivated subjects, potentially affecting the outcomes.

Only 2 studies out of 13, report the duration of their interventions to be one year, presenting a significant difference in their results [32, 39]. In contrast, one study intervention lasted for 1 month, reporting a positive effect in FoF immediately after the intervention, but after a year of follow-up, no difference was observed between the groups [41]. This result is in agreement with a recent review involving older adults living in a community where exercise interventions can reduce FoF immediately after the intervention period, but there is uncertainty in determining whether interventions have a positive effect on FoF after the interventions have ended [48]. Also, we can assume that the ideal and reliable composition and duration of physical intervention programs are elusive, in agreement with a previous review [49].

Following the above, another parameter that needs evaluation when interpreting the research results of the above-mentioned reports is the follow-up time of each intervention. Follow-up time is not studied in all reports, and when studied refutable results appear to occur [35]. The absence of supervision might lead to patient drop-out or adverse effects due to mismanagement of performed exercise [41]. Such adverse events could be partially circumvented by follow-up phone calls and regular home visits by qualified instructors that will assess the well-being and frailty phenotype of the trainees [49]. On the contrary, clinic or outdoor-organized training sessions favor adherence and social interactions of the participants but are not always feasible to be organized and maintained for longer periods [50]. In addition, the intensity of exercise should be personalized, a parameter that is not easily applicable in group-based approaches [29, 51].

Enhancing physical activity is an area in which many professionals are involved as highlighted in this systematic review, but no study presents results of interdisciplinary collaboration. Interventions yield better results when an integrated approach is used, involving all relevant health professionals including general practitioners, nurses, and specialists in palliative care [52]. The best and most cost-effective outcomes for promoting healthy aging are achieved by interdisciplinary teams working together, capturing a prognosis, and generating new intervention ideas [52].

A study that assessed FoF with a single-item question showed a lack of clarity in results immediately after interventions versus 1 year of follow-up [41]. Single-item question approaches have been challenged in detecting the degree of concern about falling compare to other assessment tools [53]. This makes the results of physical activity concerning FoF unclear. The FES-I assessment tool focuses on the individual's falls efficacy, measuring the level of concern about falling during social and physical activities [54, 55]. The ABC scale evaluates balance confidence and like the FES-I, it inquires about the individual’s assurance in their ability to carry out everyday tasks without falling [56]. Falls efficacy is related to one’s perceived ability to perform activities without falling, balance confidence refers to someone’s belief about his ability to maintain balance while performing an activity and FoF is a term that signifies concerns about the possibility of experiencing a fall [4, 8, 57]. Although FES-I and ABC are reliable for measuring self-confidence [58], it is important to recognize that equating FoF with falls efficacy and balance confidence may pose conceptual challenges, thus hindering a precise assessment of FoF [8].

The results are encouraging and recapitulate the role of physical activity in FoF reduction. Thus, it is inextricably linked to the falling propensity in the elderly and should be thoroughly assessed in attempts to focus on fall incidence reduction as previous research has demonstrated that regular exercise can be effective in preventing falls among community-dwelling older adults [59]. Such information should be taken into consideration by practitioners providing care to frail and pre-frail older adults through national or regional services to promote healthy aging. Also, given that aging inflicts a tremendous burden on state expenditures intended for older adults, specialized practitioners could benefit from such reports to better define which individuals are at greater risk within the aging population [60].

There is still a need for future studies to better address the link between FoF reduction and associated interventions, including longer follow-up periods, longer intervention duration, well-defined FοF-assessing tools, and participation of interdisciplinary teams. Also, future studies should focus more on parameters such as the combination of multiple interventions, and the possibility of home care provision by specialized practitioners. These parameters remain so far ambiguous regarding FoF reduction. In addition, it is important that future research focuses on particular aspects related to falls such as concern, fear, anxiety, balance confidence, and self-efficacy, when studying this outcome.

### Limitations

The present systematic review is subject to limitations. The search strategy was not pre-registered on PROSPERO (or equivalent). We did not include articles published in a language other than English. One study [39] uses only the ABC scale to assess FoF which may not be an effective tool for frail and pre-frail community-dwelling older adults [58]. One study [39] uses TUG to measure frailty even though it is not a common tool. It can be used as a sensitive and specific proxy for frailty and a specific proxy for pre-frailty that can be applied where the application of Fried’s criteria is not practicable [61]. There are four studies [31, 35, 40, 41] that do not specify how frailty was defined, so we cannot be very confident of these findings. This perhaps also shows the low level of research design of previous studies. Although most studies included the FES-I and ABC to assess FoF, the literature has provided more precise definitions of these concepts, emphasizing the utilization of these scales as indirect tools for evaluating fear [4, 6, 8, 56, 57]. Another limitation of the study is that reported data were retrieved from the main publication without additional contact for incomplete information with the authors. Articles with a limited explanation of intervention criteria or questionable follow-up procedures were mainly excluded from the study.

## Conclusion

The present systematic review underlines the role of physical-activity interventions on FoF reduction in frail and pre-frail older adults. It appears that interventions focusing on muscle strengthening with the combination of balance or mobility exercises are most effective in reducing FoF. Further research is needed to determine the long-term efficacy of physical-activity interventions on FoF beyond the end of the intervention period.

Our results are of great importance for healthcare professionals providing home or institutional care, supporting frail and pre-frail older adults to regain a more active and social life. In addition, the analysis of the results of this systematic review can be useful for health authorities and healthcare decision-makers to decide how to best allocate health resources and further promote the exercise of both frail and pre-frail older adults and health professionals.

### Supplementary Information

Below is the link to the electronic supplementary material.

Supplementary file1 (DOCX 33 KB)
